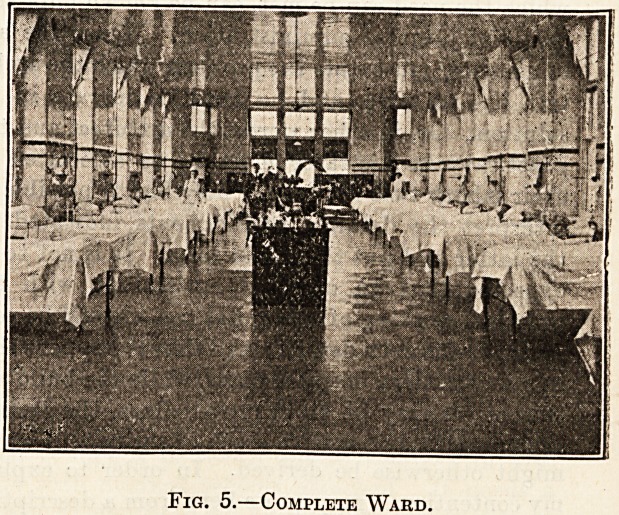# The Units of General Hospital Construction

**Published:** 1907-05-11

**Authors:** 


### HOSPITAL ADMINISTRATION.

### CONSTRUCTION AND ECONOMICS.

### THE UNITS OF GENERAL HOSPITAL CONSTRUCTION.

#### II. THE WARD UNIT.

The main principles upon which the construction of a modern hospital must proceed can, no doubt, be laid down and applied by any skilful architect. But there are details of construction in an up-to-date hospital the need of which, and the conditions under

Fig 1.—Urine Room.

which the need can be met, can be known only as a result of hospital administration. Hence the administrator must state the problem which the architect has to solve, and he must co-operate in its solution. Or, in other words, the architect and the practical administrator must be so associated in their labour that the former may find it possible to interpret the detailed requirements indicated by the latter and put them into form. It is not to be expected that an architect unfamiliar with the practical working of a hospital can himself be conversant with such details; and thus it happens that in many modern hospital plans, while the buildings themselves may be on approved lines, the lack of such detail may negative much of the benefit which might otherwise be derived. In order to explain my contention, I may quote here from a description of sanitary towers, "No urine or other excretions should be retained in vessels but should immediately be discharged by the urinal."

Now the fallacious nature of such an argument and its bearing on the fitting up of a sanitary tower, must be at once apparent to those who have any practical knowledge of hospital work, for in order to make a satisfactory examination of urine in certain classes of cases it is essential that it be collected for 12 or even 24 hours. This involves the provision of suitable storage accommodation, and by arranging the fittings of the sanitary tower as figured in the accompanying illustration, it is possible to retain urine or other excreta for the required period without incurring any risk of the odour penetrating to the ward, or causing danger or inconvenience to its inmates or attendants.

This arrangement (fig. 1) consists of a series of galvanised iron racks on which are placed the covered vessels containing the excreta, while in the wall behind are a number of openings to the external air guarded on the outside by louvre frames for ventilation, and in the inside by the iron gratings seen in the figure. By a simple arrangement these louvre frames can be opened and closed at will. Fig. 2 illustrates another arrangement for the storage of urine or excreta occupying practically the same wall space as shown in fig. 1; but in this case the racks are entirely enclosed by means of sliding iron doors. This might appear the more perfect method, but experience has proved that in practice the open method serves the purpose equally well, and is less costly in construction.

But to return to the description of the towers. In one tower the w.c.'s should be placed, and, as previously indicated, these should be completely separated by means of a brick partition extending to the roof. This tower should contain no other fittings of any kind. The windows should extend

Fig. 2.—Urine Room with Closing Doors.

to the ceiling, in order that there may be no space for foul gases to collect. The corresponding tower at the other corner contains the rack above described and illustrated, and in addition thereto a bed-pan washer, with cold water supply only, a wash-hand basin for the use of the nurse, with hot and cold water, a radiator for heating purposes, and above this radiator is fixed a galvanised iron grill where bed-pans may be slightly heated before being used.

There is also in this apartment a simple arrangement for the temporary storage of soiled linen (fig. 3). This consists of a galvanised iron ring about 15 inches in diameter, which is bolted to the wall and supports the clothes-bag. The bag is attached to the ring by a leather strap and secured by a clip. Soiled linen may be dropped into this bag and removed to the laundry without further handling by the nurse, provided she has taken due note in her laundry book of each article placed therein. When the apparatus is not in use it is unhooked and lies flat against the wall.

Leading from the cut-off passage between this

Fig. 3.—Soiled Linen Canvas Bag.

tower and the ward is a fire-escape stair. Fire-escape stairs should be so arranged that they will not cross any windows. There are two reasons for this: (1) That they will not interfere with the lighting and ventilation of any part of the building; and (2) that they may not be rendered valueless in emergency by flames escaping from such windows. The stair should be not less than four feet wide and have frequent landings, so that a patient might be removed on a stretcher, which is practically impossible with a circular stair.

In the ward unit under consideration the door, 3 feet 6 inches wide, leading to the escape stair is kept locked, and except in case of fire can only be opened by a master key. The ordinary key is placed in an enclosure in the centre of the door, glazed with red glass on the outside and clear glass on the inside. The inner glass bears the notice in bold letters, "Key of door—glass to be broken in case of fire." The key can be seen lying within. This ensures that it is always available, but that the door cannot be used for other than its legitimate purpose.

With regard to the ward itself, reference has already been made to the windows, floors, and walls. It may be mentioned here, however, that the fur-

Fig. 4.—Draught Screens.

nishings are of the simplest possible type. The beds have been specially designed for the building. The couches, tables, cabinet, and reclining chairs are all on solid rubber castors. The draught screens, illustrated (fig. 4), are also on castors, and are simple wood frames covered with a washable material,

Fig. 5.—Complete Ward.

turkey red being found very efficient and imparting a bright appearance to the ward. The covers are removable, and the frames are so light that they can be easily moved about by a nurse. The heating and ventilation of the ward will be referred to in connection with the general heating and ventilation of the building. Fig. 5 illustrates one of the wards of this unit.

(*To be continued.*)

## Figures and Tables

**Fig 1. f1:**
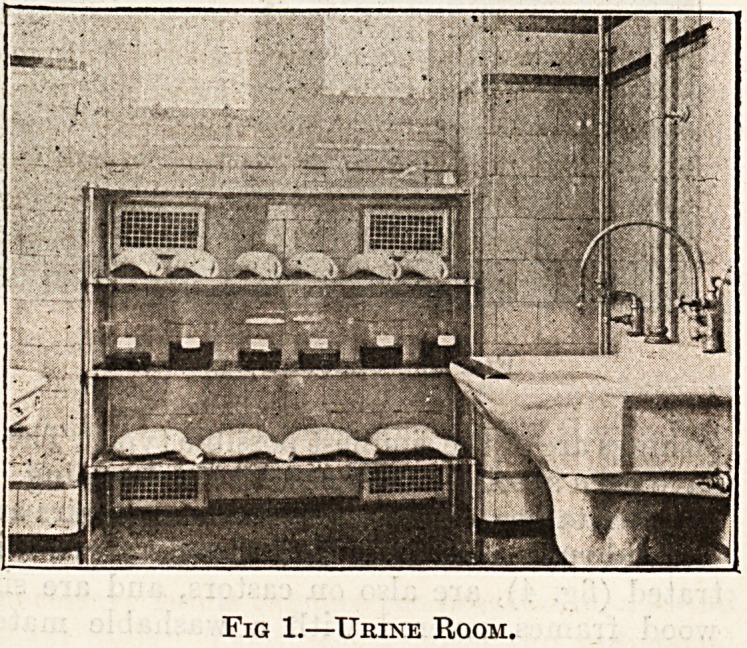


**Fig. 2. f2:**
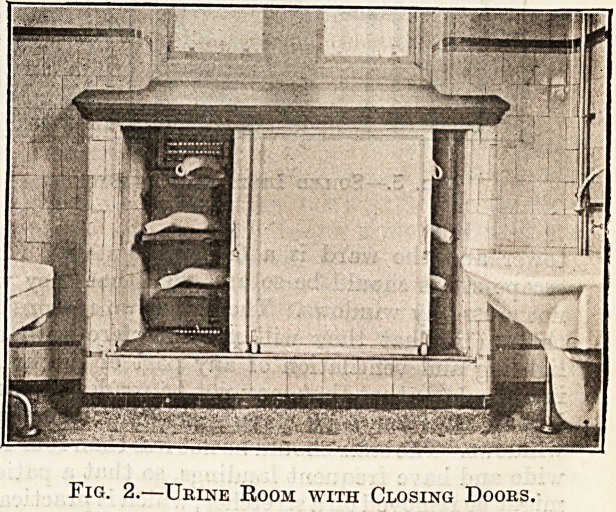


**Fig. 3. f3:**
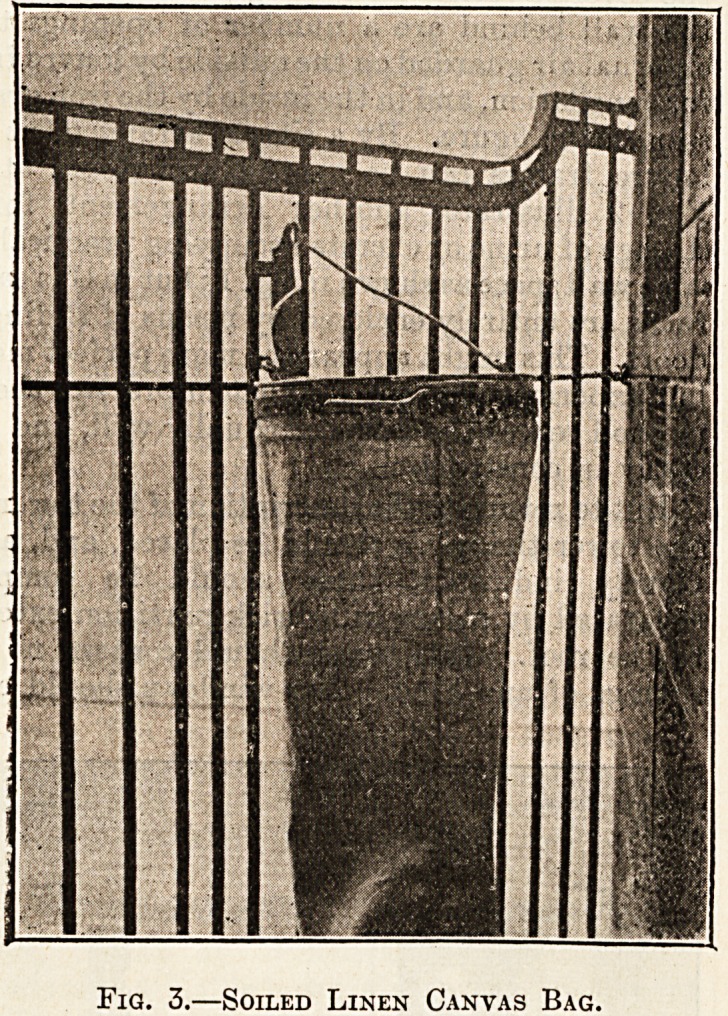


**Fig. 4. f4:**
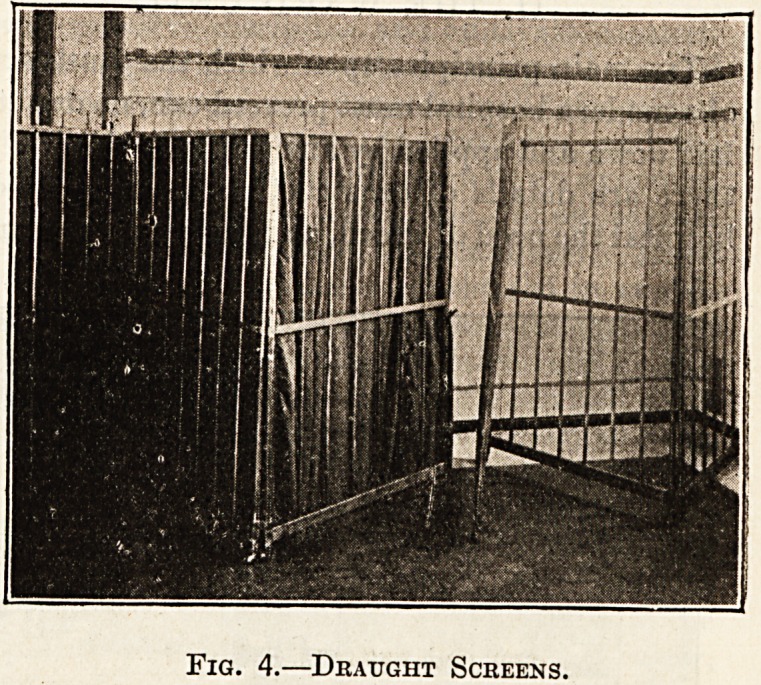


**Fig. 5. f5:**